# Assessing the relationship between body mass index and influenza-like illness risk and symptom severity among military health system beneficiaries vaccinated against influenza: a pooled prospective cohort study from a randomized trial

**DOI:** 10.3389/fpubh.2026.1874186

**Published:** 2026-07-16

**Authors:** Mofan Gu, Stephanie A. Richard, Rhonda E. Colombo, Kat Schmidt, Anuradha Ganesan, Wesley R. Campbell, Katrin Mende, Srihari Seshadri, Adam Saperstein, David E. Hrncir, Ryan C. Maves, Robert J. O'Connell, Christina E. Spooner, Mark P. Simons, Laurie A. Housel, Alan Williams, John H. Powers, Anthony Fries, Christian L. Coles, Timothy H. Burgess, Simon D. Pollett

**Affiliations:** 1 Infectious Disease Clinical Research Program, Department of Preventive Medicine and Biostatistics, Uniformed Services University of the Health Sciences, Bethesda, MD, United States; 2 The Henry M. Jackson Foundation for the Advancement of Military Medicine, Inc., Bethesda, MD, United States; 3 Department of Medicine, Uniformed Services University of the Health Sciences, Bethesda, MD, United States; 4 Madigan Army Medical Center, Tacoma, WA, United States; 5 Walter Reed National Military Medical Center, Bethesda, MD, United States; 6 Brooke Army Medical Center, San Antonio, TX, United States; 7 Immunization Healthcare Division, Defense Health Agency, Falls Church, VA, United States; 8 Department of Family Medicine, Uniformed Services University, Bethesda, MD, United States; 9 Carl R. Darnall Army Medical Center, Fort Cavazos, TX, United States; 10 Wilford Hall Ambulatory Surgical Center, Lackland Air Force Base, San Antonio, TX, United States; 11 Section of Infectious Diseases, Wake Forest University School of Medicine, Winston-Salem, NC, United States; 12 Womack Army Medical Center, Fort Bragg, NC, United States; 13 Clinical Research Directorate, Frederick National Laboratory for Cancer Research, Frederick, MD, United States; 14 US Air Force School of Aerospace Medicine, Wright-Patterson AFB, OH, United States

**Keywords:** body mass index, influenza, influenza-like illness, military health, obesity

## Abstract

**Introduction:**

Obesity prevalence is rising globally and associated with increased influenza mortality. The relationship between obesity and influenza-like-illness (ILI) incidence and symptom severity is less clear.

**Materials and methods:**

US Military Health System (MHS) beneficiaries enrolled in a randomized influenza vaccine trial received one of three influenza vaccines and were followed for ILI via weekly surveys throughout one influenza season. Participants were pooled across vaccine arms into a prospective cohort. We compared ILI risk and ILI symptom severity (Flu-PRO) across body mass index (BMI) categories using multivariable regression models.

**Results:**

Among the 8,334 participants with >50% response rates to weekly ILI surveys, 42% had an overweight-range BMI and 24% had an obesity-range BMI. The median age was 36.2, and 86% had no baseline comorbidities. Over one quarter of the participants (27%) reported at least one ILI during follow-up, with a median duration of 11 days. Participants with an overweight and obesity-range BMI were more likely to report ILIs [adjusted incidence rate ratio (aIRR) 1.18 (95% confidence interval (CI): 1.05–1.33) and 1.59 (95% CI: 1.37–1.84), respectively]. Participants with obesity also had higher risks of reporting moderate-to-severe symptoms in four Flu-PRO domains: gastrointestinal, respiratory, systemic, and throat [adjusted risk ratio (aRR) of 1.64 (95% CI: 1.01–2.70), 1.33 (95% CI: 1.05–1.67), 1.32 (95% CI: 1.06–1.63), and 1.20 (95% CI 1.02–1.40) respectively].

**Conclusions:**

Higher BMI was associated with ILI incidence and severity. These findings prompt further research into optimal strategies to reduce the impact of ILI in MHS settings and the general population.

## Introduction

Seasonal influenza remains a significant public health concern in the United States (US), infecting an estimated 47 to 82 million people with an associated 27,000 to 130,000 deaths between 2024 and 2025 (1). While the burden of severe illness is typically lower among generally healthy, highly vaccinated military populations, influenza and influenza-like illness (ILI) continue to contribute substantially to morbidity and healthcare utilization within this group (2). Classic risk factors for influenza infection and severity include age, chronic cardiorespiratory disease, neurological comorbidities, significant immunosuppression, and malignancy (3). Obesity has been increasingly understood to play a role in severe influenza, as with other respiratory illnesses such as COVID-19 (4–6). Given the increasing rates of obesity (body mass index [BMI]≥30) in the US (including in the US military), understanding the impact of obesity on influenza/ILI risk and clinical outcomes is valuable to inform clinical decision making around respiratory infections, as well as to better understand the relationship between obesity and respiratory illness outcomes (7, 8)

Interest in the potential link between obesity and influenza morbidity and mortality has grown since the 2009 A/H1N1 influenza pandemic (9). Several epidemiologic studies found positive associations between obesity and severe A/H1N1 complications (10, 11). In addition, some studies found that adults with obesity are twice as likely to develop influenza and ILI as adults with normal weight, even after vaccination (12, 13)

Current Centers for Disease Control and Prevention guidance indicate that severe obesity (BMI ≥40) is a risk factor for severe influenza (3). These BMI associated risk factors are typically derived from patients with more severe outcomes (hospitalization, death), not from patient reported outcomes (PROs) in those with outpatient illness. Moreover, the management of respiratory infections in young, healthy adults is often based on the treatment of symptoms (i.e., as an ILI syndrome), often without pathogen diagnosis nor specific treatment (14).Thus, there remain gaps in our understanding of the impact of obesity on milder outpatient influenza and the syndrome of ILI.

Annual influenza vaccination was mandatory for active and reserve component US service members throughout the study period; the role of obesity in influenza and ILI outcomes, including PROs, in this highly vaccinated population is unclear. Studying obesity and the risk of influenza and ILI outcomes is of particular interest to the US military because influenza infection, and ILI more broadly, are leading causes of duty and training days lost in the active component (15). Close living quarters, frequent travel, and operational or training-associated stress potentially increase risk of exposure to and transmission of influenza and ILI (14, 16). According to a recent study, roughly 70% of active-duty personnel had an overweight (25–29.9) or obese (≥30) BMI-range, and the prevalence of obesity is increasing (17).

In this study, we examined (1) whether higher BMI is associated with increased risk of ILI, and (2) whether higher BMI predicts more severe ILI symptoms, including quantitative symptoms elicited by a structured PRO tool (Flu-PRO), among a large prospective cohort of vaccinated Military Health System (MHS) beneficiaries. We also explored (3) whether BMI is associated with the risk of infection by specific ILI-associated pathogens, including influenza, rhino/enterovirus, human metapneumovirus (HMPV), parainfluenza virus (PIV), and respiratory syncytial virus (RSV).

## Materials and methods

**Figure 1:**
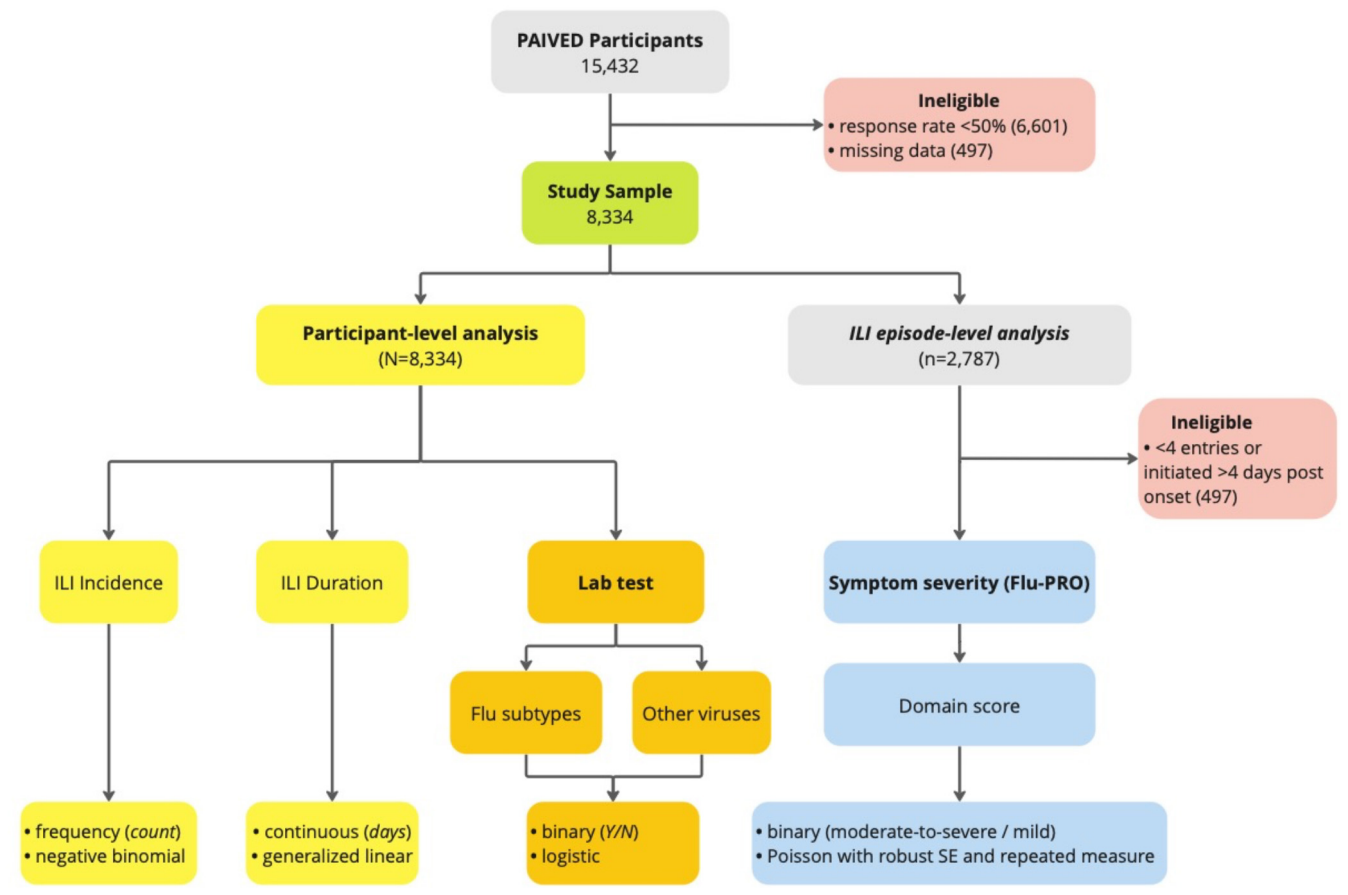
Flow diagram of participant selection and analytic outcomes in PAIVED study participants who reported BMI information and had a survey response rate of 50% and higher (n = 8,334). The endpoints and methods of assessments for ILI (incidence, illness duration, and symptom severity) and underlying lab-confirmed pathogens (including influenza subtypes) are depicted.

The Pragmatic Assessment of Influenza Vaccine Effectiveness in the Department of Defense (PAIVED) study was a randomized clinical trial that evaluated the relative effectiveness of three FDA-licensed influenza vaccines among MHS beneficiaries aged 18 years or older. Participants were randomized to receive one of three study influenza vaccines and followed during the influenza season in which they enrolled. The study was conducted across four consecutive influenza seasons (2018–2022) (18). During the influenza season, participants received weekly surveys in which they were asked if they had experienced ILI symptoms during the past week. Participants who met the ILI case definition were asked to complete an online symptom severity questionnaire for seven days, and schedule two ILI visits, approximately 28 days apart, to provide ILI information (e.g., medication, hospitalization, work absence) (18). A nasal swab was collected during the acute visit, which was scheduled as soon as possible after the ILI was confirmed by study staff. Our analysis included PAIVED participants who responded to at least half of the ILI surveys and all required demographic variables (Figure 1). BMI was calculated using weight and height reported at enrollment or, if that was missing, anthropometric measures collected at the most recent medical visit around time of enrollment (abstracted from the electronic medical record): $BMI=\frac{Weight\;\left(kg\right)}{{height\;\left(m\right)}^{2}}$, and stratified into three categories: normal/underweight (BMI < 25 kg/m^2^), overweight (25 ≤ BMI < 30 kg/m^2^), and obese (BMI ≥30 kg/m^2^). Due to the small number of underweight participants (47, 0.6%), we combined them with the normal weight group for all analyses. The primary results of the PAIVED trial indicated no difference in clinical effectiveness between vaccines, allowing for pooling of the vaccine groups for this analysis (18).

### Outcome definition

We examined four categories of ILI outcomes: incidence, duration, severity, and laboratory-confirmed respiratory infections. Unless otherwise specified, outcomes were assessed at the participant level. ILI incidence was defined as the total count of study-identified ILI episodes for each participant during the influenza season. ILI duration was defined as the maximum self-reported duration during the season, based on participant reports at acute and convalescent visits. Symptom severity was measured prospectively using the Influenza Patient-Reported Outcome (Flu-PRO) questionnaire (19). The Flu-PRO questionnaire asks participants to rate the severity of 34 symptoms over the past 24 h, using a 5-point Likert-type scale ranging from ‘Not at all' to ‘Very much' (19). For five of these items—sneezing, coughing, coughing up mucus or phlegm, vomiting, and diarrhea—responses are recorded as symptom frequency rather than severity, using response options of “Never”, “Rarely”, “Sometimes”, “Often”, or “Always” for respiratory symptoms and “0 times”, “1 time”, “2 times”, “3 times”, or “4 or more times” for vomiting and diarrhea (19). Higher scores indicate more severe symptoms. Symptoms are categorized into six domains: nose, throat, eye, systemic, respiratory, and gastrointestinal; a seventh domain, sense, was added to the questionnaire in May 2020 to capture loss of sense of smell and/or taste (Supplemental Table 1). Additional self-reported illness characteristics, including days with limited activity, days missed from work, days with fever, and days using fever-reducing medications, were collected and summarized descriptively. Participants who reported an ILI were asked to fill out the Flu-PRO questionnaire daily for seven days. Mean overall and domain-specific scores were calculated for each daily entry and the maximum score over the seven-day period was calculated. Total scores were calculated without the sense domain (added in 2020) for comparability across the study timeframe. The maximum Flu-PRO scores were dichotomized into 2 categories: “little to no symptoms” (scores < 2) and “moderate-to-severe symptoms” (scores ≥2). Flu-PRO–derived illness characteristics, including days with fever, use of fever-reducing medications, days with limited activity, and days missed from work, were summarized descriptively. Flu-PRO outcomes were assessed at the ILI episode level. ILI episodes with fewer than four days of Flu-PRO entries, or Flu-PRO entries initiated more than four days post symptom onset, were excluded from our analysis. Lab-confirmed respiratory infections were identified using reverse transcription polymerase chain reaction (RT-PCR) from a study-collected nasal swab during the ILI episode (18). For these outcomes, a value of one indicates a positive test, and a value of zero indicates a negative result (rather than not being tested). Multiplex RT-PCR assays (NxTAG Respiratory Pathogen Panel, Diasorin/Luminex, Austin, TX) were used to detect the following viruses: influenza (overall and by subtype [A, A/H1N1, A/H3N2, A/untyped, and B]), adenovirus, bocavirus, human coronavirus, human metapneumovirus (HMPV), parainfluenza virus (PIV), rhino/enterovirus, respiratory syncytial virus (RSV), and Severe Acute Respiratory Syndrome Coronavirus 2 (SARS-CoV-2) (18).

### Statistical analysis

Participant characteristics and outcomes were compared across BMI strata using Chi-square tests or Fisher's exact tests, as appropriate, for categorical variables and Kruskal-Wallis tests for continuous variables. Regression models were then used to estimate associations between BMI strata and each outcome, with the unit of analysis defined according to outcome type. Participant-level analyses were used to estimate the incidence of ILI episodes using negative binomial regression, which was selected due to mild overdispersion in ILI episode counts. ILI duration was assessed using generalized linear regression, and the risk of specific laboratory-confirmed respiratory infections was estimated using logistic regression. Analyses of lab-confirmed respiratory infections were restricted to participants who had a tested respiratory specimen. Episode-level analyses were used to estimate the risk of moderate-to-severe symptom severity by Flu-PRO domain using Poisson regression with robust standard errors, accounting for repeated ILI episodes within participants. The analytic flow is shown in Figure 1.

Potential confounders were identified *a priori* via directed acyclic graphs (DAGs) and included sex, age at enrollment, race, ethnicity, highest education level, activity level (self-reported ‘active' or ‘sedentary'), smoking status (never, former, or current smoker), Deyo-Charlson Comorbidity Index (DCCI), Department of War (DoW) affiliation (Army, Navy, Air Force, or other), military status (active duty, retired, or dependent), and season of enrollment (Supplemental Figure 1) (20). The DCCI was calculated using healthcare encounter records in the year prior to enrollment in PAIVED (21, 22). Multicollinearity was assessed before inclusion of DAG variables into a final model. The same set of covariates was used in adjusted models for ILI incidence, ILI duration, symptom severity, and laboratory-confirmed respiratory virus outcomes. To assess the robustness of our findings, we also conducted sensitivity analyses examining effect modification of age and comorbidities in the main model. In addition, we examined parsimonious models selected via stepwise selection procedures, restricting inclusion to a limited number of covariates. For outcomes of lab-confirmed influenza, adenovirus, bocavirus, HMPV, PIV, and RSV, only parsimonious models were used due to the relatively small number of confirmed cases.

Statistical analyses were conducted using SAS studio (SAS Institute INC., Cary, NC) and data visualization was performed in RStudio (Posit Software, PBC, Boston, MA) using R version 4.5.1 (R Foundation for Statistical Computing, Vienna, Austria). We used *p* < 0.05 as the level of statistical significance.

### Ethics

This study was conducted following good clinical practice and according to the Declaration of Helsinki guidelines. PAIVED (IDCRP-120) was approved by the Uniformed Services University Institutional Review Board. All study participants provided written informed consent when enrolled in the study. The PAIVED clinical trial is registered at ClinicalTrials.gov (identifier NCT03734237) (23).

## Results

Among the 8,334 MHS beneficiaries included in our analysis (Figure 1, Table 1), 2,852 (34.2%) had a normal/underweight-range BMI, 3,512 (42.1%) had an overweight-range BMI, and 1,970 (23.6%) had an obese-range BMI. Overall, participants in our study population were predominantly non-Hispanic, White, active duty, non-smoker, and living an active lifestyle. Over a quarter of the participants [2,275 (27.3%)] reported at least one ILI during the season in which they were enrolled (Table 2); 1,825 (21.9%) reported one, 388 (4.7%) reported two, and 62 (0.7%) reported three ILIs. The median reported ILI duration was 11.0 days. Crude incidence rate ratios (IRRs) were 1.08 (95% CI: 0.98–1.18) for participants with an overweight-range BMI and 1.27 (95% CI: 1.15–1.41) for participants with obesity. After adjustment for covariates, the corresponding adjusted IRRs (aIRRs) were 1.18 (95% CI: 1.05–1.33) and 1.59 (95% CI: 1.37–1.84), indicating an increased risk of ILI among participants with an overweight or obesity-range BMI compared with participants with a normal weight or underweight-range BMI (Supplemental Table 2). Crude point estimates suggested longer ILI duration among participants with obesity compared with participants with a normal weight or underweight-range BMI (by 0.96 days, 95% CI: 0.08–1.83), but this association was attenuated and no longer statistically significant after adjustment (by 0.38 days, 95% CI: −0.58 to 1.33) (Supplemental Table 3). No notable differences in ILI duration were observed for participants meeting overweight criteria.

**Table 1:** Characteristics of PAIVED study participants selected for analysis, by body mass index (BMI) category. All variables were statistically significantly different across the BMI categories (p < 0.001) based on Chi-squared tests (categorical variables) and Kruskal-Wallis test (age).

		Participants, no. (%)^a^			
Characteristic	Level	Normal/underweight (*N* = 2,852)	Overweight (*N* = 3,512)	Obese (*N* = 1,970)	Total (*N* = 8,334)
Sex	Male	1,420 (49.8%)	2,323 (66.1%)	1,259 (63.9%)	5,002 (60.0%)
Age	Median (IQR)	31.0 (23.7,41.8)	36.2 (28.4,47.7)	44.5 (34.4,56.4)	36.2 (27.5,48.8)
Race	White	2,190 (76.8%)	2,625 (74.7%)	1,374 (69.7%)	6,189 (74.3%)
	Black or African American	184 (6.5%)	374 (10.6%)	355 (18.0%)	913 (11.0%)
	Asian	267 (9.4%)	202 (5.8%)	47 (2.4%)	516 (6.2%)
	Multiple	133 (4.7%)	169 (4.8%)	90 (4.6%)	392 (4.7%)
	Other	78 (2.7%)	142 (4.0%)	104 (5.3%)	324 (3.9%)
Ethnicity	Hispanic	312 (10.9%)	477 (13.6%)	381 (19.3%)	1,170 (14.0%)
Educational status	High school or below	882 (30.9%)	850 (24.2%)	460 (23.4%)	2,192 (26.3%)
	Associates	252 (8.8%)	485 (13.8%)	474 (24.1%)	1,211 (14.5%)
	Bachelors	578 (20.3%)	798 (22.7%)	473 (24.0%)	1,849 (22.2%)
	Higher (e.g., Masters, PhD)	1,140 (40.0%)	1,379 (39.3%)	563 (28.6%)	3,082 (37.0%)
Activity level	Active	2,667 (93.5%)	3,151 (89.7%)	1,471 (74.7%)	7,289 (87.5%)
Smoking status	Current smoker	113 (4.0%)	156 (4.4%)	93 (4.7%)	362 (4.3%)
	Former smoker	201 (7.0%)	403 (11.5%)	380 (19.3%)	984 (11.8%)
	Non-smoker	2,538 (89.0%)	2,953 (84.1%)	1,497 (76.0%)	6,988 (83.8%)
Charlson comorbidity index	0	2,638 (92.5%)	3067 (87.3%)	1,475 (74.9%)	7,180 (86.2%)
	1–2	154 (5.4%)	346 (9.9%)	366 (18.6%)	866 (10.4%)
	3–4	14 (0.5%)	46 (1.3%)	75 (3.8%)	135 (1.6%)
	5 and higher	46 (1.6%)	53 (1.5%)	54 (2.7%)	153 (1.8%)
DoW affiliation	Army	1,225 (43.0%)	1,764 (50.2%)	1,005 (51.0%)	3,994 (47.9%)
	Navy	1,113 (39.0%)	1,067 (30.4%)	512 (26.0%)	2,692 (32.3%)
	Air force	446 (15.6%)	565 (16.1%)	382 (19.4%)	1,393 (16.7%)
	Other	68 (2.4%)	116 (3.3%)	71 (3.6%)	255 (3.1%)
Military status	Active duty	2,251 (78.9%)	2,599 (74.0%)	901 (45.7%)	5,751 (69.0%)
	Retired	247 (8.7%)	527 (15.0%)	627 (31.8%)	1,401 (16.8%)
	Dependent	354 (12.4%)	386 (11.0%)	442 (22.4%)	1,182 (14.2%)
Season	2018–2019	128 (4.5%)	197 (5.6%)	236 (12.0%)	561 (6.7%)
	2019–2020	996 (34.9%)	1,239 (35.3%)	718 (36.4%)	2,953 (35.4%)
	2020–2021	671 (23.5%)	800 (22.8%)	410 (20.8%)	1,881 (22.6%)
	2021-2022	1,057 (37.1%)	1,276 (36.3%)	606 (30.8%)	2,939 (35.3%)

IQR, interquartile range; DoW, Department of War. ^a^BMI was stratified into three categories: normal/underweight (BMI < 25), overweight (25 ≤ BMI < 30), and obese (BMI ≥30); All values are *N* (%), unless otherwise specified.

**Table 2:** Outcome measures, including influenza-like-illness (ILI) (incidence and duration) and underlying lab-confirmed viruses among a subset of PAIVED study participants, by body mass index (BMI) category. Analyses of lab-confirmed respiratory infections were restricted to participants who had a respiratory specimen tested.

	Participants, no. (%)^a^				
Outcome	Normal/underweight (*N* = 2,852)	Overweight (*N* = 3,512)	Obese (*N* = 1,970)	Total (*N* = 8,334)	*P* ^b^
ILI incidence^b^					
1	577 (20.2%)	758 (21.6%)	490 (24.9%)	1,825 (21.9%)	0.001
2	130 (4.6%)	160 (4.6%)	98 (5.0%)	388 (4.7%)	0.81
3	11 (0.4%)	25 (0.7%)	26 (1.3%)	62 (0.7%)	0.004
Duration (days; median, IQR)^c^	11.0 (8.0, 15.0)	11.0 (8.0, 15.0)	12.0 (8.0, 16.0)	11.0 (8.0, 15.0)	0.78
Lab-confirmed infection	(*N* = 641)	(*N* = 848)	(*N* = 549)	(*N* = 2038)	
Any respiratory virus^b^	338 (52.7%)	447 (52.7%)	265 (48.3%)	1,050 (51.5%)	0.21
SARS-CoV-2^b, d^	115 (35.7%)	164 (38.0%)	79 (30.9%)	358 (35.4%)	0.17
Rhino/enterovirus^b^	130 (20.3%)	139 (16.4%)	94 (17.1%)	363 (17.8%)	0.13
Human coronavirus^b^	54 (8.4%)	77 (9.1%)	52 (9.5%)	183 (9.0%)	0.81
Influenza (any)^b^	37 (5.8%)	48 (5.7%)	24 (4.4%)	109 (5.3%)	0.49
A^b^	23 (3.6%)	33 (3.9%)	17 (3.1%)	73 (3.6%)	0.73
A/H1N^b^	16 (2.5%)	20 (2.4%)	11 (2.0%)	47 (2.3%)	0.85
A/H3N2^e^	5 (0.8%)	8 (0.9%)	4 (0.7%)	17 (0.8%)	0.90
A/untyped^e^	2 (0.3%)	5 (0.6%)	2 (0.4%)	9 (0.4%)	0.69
B^b^	14 (2.2%)	16 (1.9%)	7 (1.3%)	37 (1.8%)	0.49
RSV^b^	12 (1.9%)	25 (3.0%)	14 (2.6%)	51 (2.5%)	0.42
HMPV^b^	17 (2.7%)	14 (1.7%)	14 (2.3%)	45 (2.2%)	0.35
PIV^b^	6 (0.9%)	9 (1.1%)	10 (1.8%)	25 (1.2%)	0.33
Bocavirus^e^	2 (0.3%)	10 (1.2%)	8 (1.5%)	20 (1.0%)	0.10
Adenovirus^e^	1 (0.2%)	5 (0.6%)	2 (0.4%)	8 (0.4%)	0.41

HMPV, human metapneumovirus; PIV, parainfluenza virus; RSV, respiratory syncytial virus; SARS-CoV-2, Severe Acute Respiratory Syndrome Coronavirus 2. ^a^BMI was stratified into three categories: normal/underweight (BMI < 25), overweight (25 ≤ BMI < 30), and obese (BMI≥30). ^b^Chi-sq test was used to test for statistical significance. ^c^Kruskal-Wallis test was used to test for statistical significance. ^d^Total N for SARS-CoV-2 testing was 1010 (322 for normal/underweight, 432 for overweight, and 256 for obese). ^e^Fisher's exact test was used to test for statistical significance.

**Figure 2:**
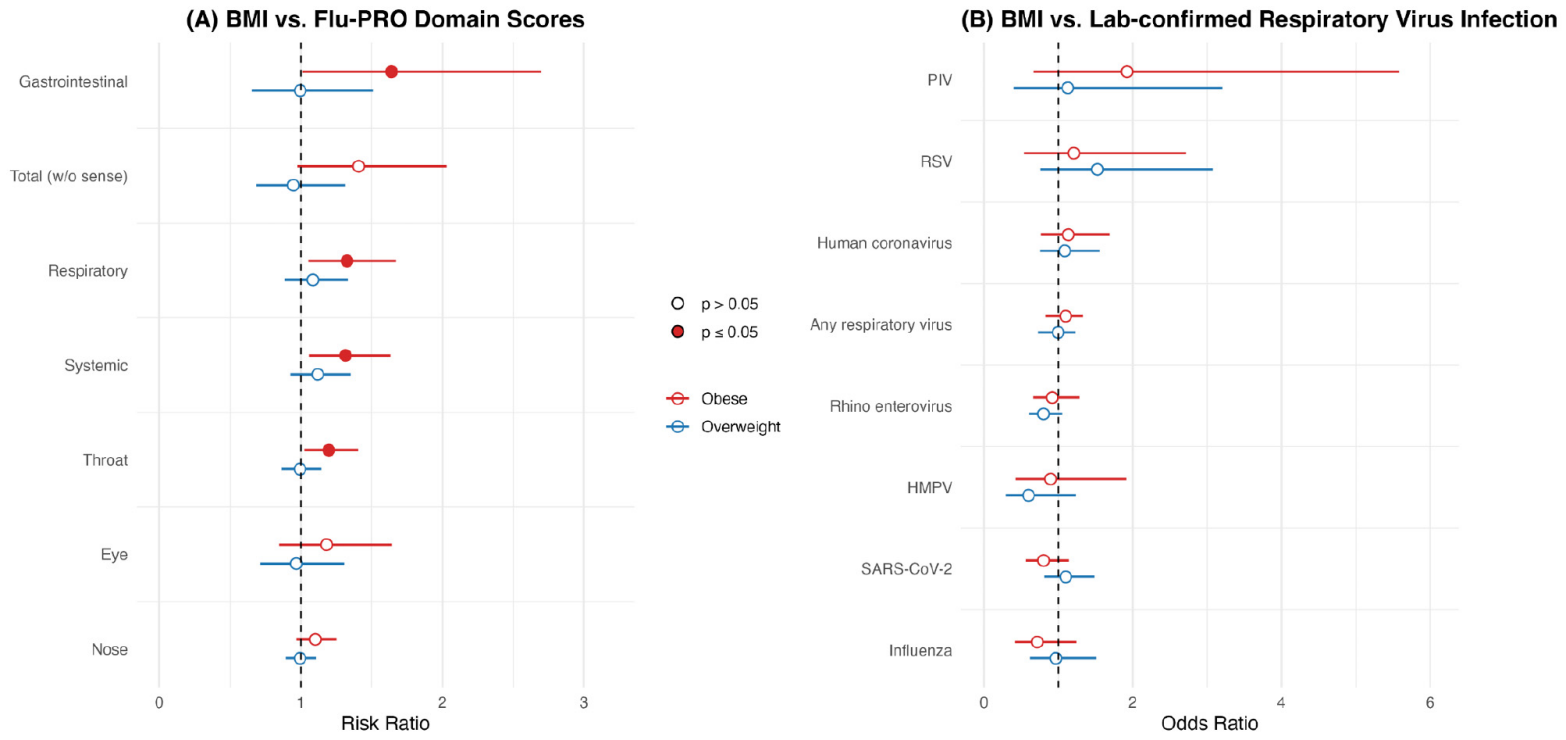
Adjusted associations between BMI category and **(A)** moderate-to-severe ILI symptom severity measured by Flu-PRO and **(B)** lab-confirmed respiratory virus infection, using normal/underweight BMI as the reference group. Risk ratios (aRR) were estimated using Poisson regression with robust variance, and odds ratios (aOR) were estimated using logistic regression. Each model was adjusted for confounders identified via directed acyclic graphs. Analyses of lab-confirmed infections were performed separately for each virus type; parsimonious models were used for lab-confirmed influenza, adenovirus, bocavirus, HMPV, PIV, and RSV. Adenovirus and bocavirus were excluded due to small case numbers. BMI, body mass index; Flu-PRO, influenza patient-reported outcome; HMPV, human metapneumovirus; PIV, parainfluenza virus; RSV, respiratory syncytial virus; SARS-CoV-2, severe acute respiratory syndrome coronavirus 2.

Moderate-to-severe symptoms as measured using Flu-PRO (Table 3) were more common in participants with an overweight or obesity-range BMI than in participants with a normal weight or underweight-range BMI. Specifically, a higher percentage of participants with obesity reported moderate to severe symptoms in the respiratory, systemic, nose, and throat domains compared to participants with a normal weight or underweight-range BMI. In addition, functional illness characteristics suggested greater overall illness burden among participants with obesity. Participants with obesity reported a longer median duration of limited activity and more days with fever and use of fever-reducing medications compared with participants with a normal weight or underweight-range BMI, while days missed from work were similar across BMI categories (Table 3). Based on adjusted Poisson regression models, participants with obesity were more likely to report moderate-to-severe gastrointestinal [adjusted risk ratios (aRRs) of 1.64 (95% CI: 1.01–2.70)], respiratory [1.33 (95% CI: 1.05–1.67)], systemic [1.32 (95% CI: 1.06–1.63)], and throat [1.20 (95% CI 1.02–1.40)] symptoms compared to participants with a normal weight or underweight-range BMI (Figure 2A, Supplemental Table 4), whereas no statistically significant differences in moderate-to-severe symptoms were associated with overweight.

**Table 3:** Frequency of moderate to severe influenza-like-illness (ILI) symptoms (maximum Flu-PRO score ≥2), and selected ILI characteristics, by ILI symptom domain and body mass index (BMI) category among PAIVED study participants selected for analysis who reported ILI.

	Episodes, no. (%)^a^				
Outcome	Normal/underweight (*N* = 670)	Overweight (*N* = 886)	Obese (*N* = 562)	Total (*N* = 2118)	*p*
Domain [*N* (%)]^b^					
Eye	76 (11.3%)	102 (11.5%)	94 (16.7%)	272 (12.8%)	0.002
Gastrointestinal	40 (6.0%)	46 (5.2%)	49 (8.7%)	135 (6.4%)	0.02
Nose	326 (48.7%)	426 (48.1%)	293 (52.1%)	1045 (49.3%)	0.19
Respiratory	131 (19.6%)	186 (21.0%)	159 (28.3%)	476 (22.5%)	< 0001
Systemic	151 (22.5%)	213 (24.0%)	164 (29.2%)	528 (24.9%)	0.007
Throat	243 (36.3%)	312 (35.2%)	245 (43.6%)	800 (37.8%)	0.003
Total^c^	64 (9.6%)	78 (8.8%)	84 (14.9%)	226 (10.7%)	< 0001
Sense^d^	40 (12.2%)	60 (13.3%)	45 (17.6%)	145 (14.1%)	0.07
ILI characteristics [median (IQR)]^e^					
Days with limited activity	4.0 (2.0, 7.0)	4.0 (2.0, 7.0)	5.0 (3.0, 7.0)	4.0 (2.0, 7.0)	0.0003
Days missed work	0.0 (0.0, 2.0)	0.0 (0.0, 3.0)	0.0 (0.0, 3.0)	0.0 (0.0, 2.0)	0.15
Days with fever	2.0 (0.0, 3.0)	2.0 (1.0, 3.0)	2.0 (1.0, 4.0)	2.0 (1.0, 4.0)	< 0001
Days on fever reducer	2.0 (0.0, 5.0)	3.0 (1.0, 5.0)	3.0 (1.0, 5.0)	3.0 (1.0, 5.0)	< 0001

^a^BMI was stratified into three categories: normal/underweight (BMI < 25), overweight (25 ≤ BMI < 30), and obese (BMI ≥30). ^b^Chi-square test was used to test for statistical significance. ^c^The total Flu-PRO score used in this analysis did not include the sense questions which were added in mid-2020. ^d^The sense questions were added to the Flu-PRO survey in mid-2020; therefore, the *N* for these questions is: Normal/underweight *N* = 396, Overweight *N* = 522, Obese *N* = 291, Total *N* = 1029. ^e^Kruskal-Wallis test was used to test for statistical significance.

Among participants who reported ILIs, at least one respiratory virus was detected in over half (51.5%) of the 2,038 participants with laboratory test results (Table 2). SARS-CoV-2 was detected in the highest percentage of tested participants (358; 35.4% of 1,010 participants tested for SARS-CoV-2), followed by rhino/enterovirus (363; 17.8%), seasonal human coronavirus (183; 9.0%), and influenza (109; 5.3%) among the 2,038 participants with non–SARS-CoV-2 test results. We observed no clear pattern of differential risk of specific respiratory viruses across BMI strata (Figure 2B, Supplemental Figure 1).

Results from effect modification analyses suggested evidence of interaction between BMI category and age in the primary ILI incidence model (Type III *p* = 0.04). Age-stratified estimates suggested that the association between obesity and ILI incidence was strongest among participants aged 18–39 years (IRR = 1.38, 95% CI: 1.20–1.59), whereas estimates were smaller and not statistically significant among participants aged 40–59 years (IRR = 1.12, 95% CI: 0.92–1.37) and ≥60 years (IRR = 1.29, 95% CI: 0.85–1.95). No evidence of effect modification by comorbidity burden was observed (Type III *p* = 0.17). Results from sensitivity analyses using parsimonious models (limited to two covariates based on stepwise selection) are presented in Supplemental Table 5. When compared to the full models, estimates for ILI incidence and ILI duration were slightly attenuated but remained directionally consistent. Findings for Flu-PRO domains were generally similar, with some symptom domains (e.g., eyes, respiratory) showing slightly stronger associations. For respiratory virus outcomes, estimates were similar with minor shifts for certain outcomes (e.g., all respiratory viruses and rhino/enterovirus in participants with obesity), but the estimates remained close to the null and not statistically significant. Overall, the sensitivity analyses support the robustness of the primary findings.

## Discussion

Our results support the independent role of BMI in both ILI susceptibility and symptom severity, but not ILI duration, in a cohort of influenza-vaccinated individuals. We observed increased ILI incidence with higher BMI strata, a trend that persisted after the inclusion of potential confounders. Our results align with previous studies which identified an association between obesity and ILI incidence in a vaccinated cohort and in the general US population (11–13).

ILI symptom severity progressively increased across BMI categories, from participants with normal weight or underweight to participants with obesity. The trend was consistently observed in all individual symptom domains. After adjusting for covariates, participants with obesity experienced significantly higher odds of moderate-to-severe ILI symptoms when compared to participants with normal weight or underweight. Consistent with higher Flu-PRO-defined symptom severity, participants with obesity also reported greater functional illness burden, including more days of limited activity and fever, supporting the interpretation that observed differences reflect broader functional illness impact.

While we observed associations between obesity and ILI risk and severity, we did not observe statistically significant associations with laboratory-confirmed respiratory virus outcomes, including influenza, and therefore our findings should be interpreted primarily in the context of ILI rather than confirmed influenza infection. Still, influenza in animal and human respiratory infection model data offers potential biological rationale for our observed epidemiological associations between obesity and ILI syndrome risk. For example, the obese host exhibits chronic low-grade inflammation and impaired cell-mediated and mucosal immunity to influenza, both of which contribute to delayed and weakened viral defense (7, 24–30). Additionally, obesity is associated with exaggerated pro-inflammatory responses from imbalanced leptin and adiponectin levels, as well as uncontrolled and aggressive viral replication (30–32).

Interestingly, effect modification analyses suggested that the association between obesity and ILI incidence may be stronger among younger participants. Although this finding should be interpreted cautiously given its exploratory nature, it may suggest that obesity-related differences in susceptibility to ILI respiratory illness are more apparent in younger adults. Further study is required to replicate this finding.

Our study had multiple strengths, including prospective active surveillance for ILI in a trial setting. Prior studies which used healthcare encounters may have been biased, in that only cases with more pronounced symptoms or high risk comorbidities were recommended for testing (13, 33). Another strength was the comprehensive prospective assessment of ILI and potential underlying etiologies using complementary data sources, including self-reported ILI episodes, RT-PCR of nasal swabs collected from participants who reported ILI, and multi-domain symptom severity measures obtained using the validated Flu-PRO instrument.

One limitation of this analysis is the potential misclassification of overweight and obesity using BMI, particularly in a population with a high proportion of active duty military service members. In military populations, BMI may overestimate adiposity among highly fit individuals due to greater muscle mass, yet it remains a practical and generally reliable indicator of body composition and cardiometabolic risk at the population level. A direct measurement (i.e., waist circumference and waist-to-hip ratio) might potentially reduce misclassification; however, some studies show that BMI is highly correlated with fat mass (*r* = 0.93–0.94) (34–37). Future studies would benefit from multiple measures of overweight and obesity.

In addition, the generalizability of our study beyond military populations is uncertain, and the effect of underweight on ILI risk could not be examined due to insufficient sample size in that stratum. As this analysis relied on patient-reported symptom data, reporting bias is possible. Finally, BMI categories were not statistically significant associated with specific respiratory viruses; however, there were low numbers of some of the lab-confirmed viruses which may have impacted our ability to detect a difference.

BMI data are easily obtainable, and our findings may have implications for risk stratification. Additionally, our findings may inform research of preventive efforts targeting metabolic health and weight management among individuals at risk for respiratory illness (38, 39). Further research which examines if such obesity countermeasures reduce the risk and outcomes of influenza and ILI is warranted. Our results also highlight the need for further research to understand the immunological differences in patients with obesity, how obesity may influence vaccine response, antiviral effectiveness, and illness outcomes for influenza and other respiratory pathogens.

In conclusion, our study used quantitative methods to assess the patient-reported impact of influenza or ILI among vaccinated MHS beneficiaries, using comprehensive and validated outcome measures from a large prospective cohort. The findings indicate that obesity is associated with increased ILI risk and symptom severity. Our study provides evidence to support patient counseling about metabolic health risks and clinical prognostication, and prompts further important research to reduce the infectious morbidity associated with obesity.

## Data Availability

The data analyzed in this study is subject to the following licenses/restrictions: The data that support the findings of this study are available from the United States Defense Health Agency. Restrictions apply to the availability of these data, which were used under federal Data User Agreements for the current study and so are not publicly available. Full protocol accessible here: clinicaltrials.gov/study/NCT03734237 Data for this study are held by the Infectious Disease Clinical Research Program (IDCRP), headquartered at the Uniformed Services University of the Health Sciences (USU), Department of Preventive Medicine and Biostatistics. Restrictions apply to the availability of these data, which were used under federal Data User Agreements for the current study, and so are not publicly available. Upon request, limited data set(s) may be available, subject to regulatory review and approval. Inquiries may be sent to: Address: 6720B Rockledge Drive, Suite 340, Bethesda, MD 20817. contactus@idcrp.org. Full protocol accessible here: clinicaltrials.gov/study/NCT03734237. Requests to access these datasets should be directed to IDCRP, contactus@idcrp.org.

## Author disclaimer

The views expressed are those of the authors and do not reflect the official policy of the Department of the Army, Department of the Navy, the Department of the Air Force, the Department of War, the US Government, US Army Medical Dept, US Army Office of the SG, BAMC, MAMC, WRNMMC, WAMC, USUHS, DHA, and the Henry M. Jackson Foundation for the Advancement of Military Medicine, Inc. (HJF). Mention of trade names, commercial products, or organizations does not imply endorsement by the US Government. Investigators followed human subjects' protection 45CFR46 policies.
