# Supplementary material for: Assessing the relationship between body mass index and influenza-like illness risk and symptom severity among military health system beneficiaries vaccinated against influenza: a pooled prospective cohort study from a randomized trial
Source: Front Public Health. 2026 Jul 16;14:1874186. doi: 10.3389/fpubh.2026.1874186 (PMC13422495; doi:10.3389/fpubh.2026.1874186)
Supplement: Supplementary file 1 [file Supplementary_file_1.docx]

**Supplement**

**Supplemental Table 1.** Symptoms included in Flu-PRO questionnaire, by domain

| **Domain** | **Symptoms** |
| --- | --- |
| Nose | runny nose, stuffy nose, sneezing^a^, sinus pressure |
| Throat | scratchy throat, sore throat, difficulty swallowing |
| Eye | teary eyes, sore eyes, eyes sensitive to light |
| Systemic | body aches, weak, chills, felt cold, felt hot, sweating, headache, head congestion, felt dizzy, sleeping more |
| Respiratory | trouble breathing, chest congestion, chest tight, dry cough, wet cough, coughing^a^, coughed up mucus^a^ |
| Gastrointestinal (GI) | felt nauseous, stomachache, lack of appetite, vomit^b^, diarrhea^b^ |
| Sense | altered smell, altered taste |
| Total (without sense) | nose + throat + eye + systemic + respiratory + GI |
| Total | nose + throat + eye + systemic + respiratory + GI + sense |

^a^Scale: never, rarely, sometimes, often, always

^b^Scale: 0, 1, 2, 3, 4 or higher

**Supplemental Table 2.** Crude and adjusted negative binomial regression model estimates to identify association between covariates and number of ILI episodes reported among PAIVED study participants selected for analysis

| **Characteristic** | **Crude^a^** | | **Adjusted** | |
| --- | --- | --- | --- | --- |
|  | **IRR (95% CI)** | **p** | **aIRR (95% CI)** | **p** |
| Activity Level | |  |  |  |
| Sedentary | 1.11 (0.99 1.24) | 0.07 | 1.07 (0.91, 1.24) | .42 |
| Active | ref |  | ref |  |
| Age | 0.99 (0.99, 1.00) | <.0001 | 0.99 (0.99, 1.00) | .01 |
| BMI |  |  |  |  |
| Overweight | 1.08 (0.98, 1.18) | 0.11 | 1.18 (1.05, 1.33) | .01 |
| Obese / Severely Obese | 1.27 (1.15, 1.41) | <.0001 | 1.59 (1.37, 1.84) | <.0001 |
| Normal / Underweight | ref |  | ref |  |
| CCI |  |  |  |  |
| 1-2 | 0.95 (0.84, 1.08) | 0.46 | 1.02 (0.85, 1.21) | .85 |
| 3-4 | 0.97 (0.71, 1.32) | 0.83 | 1.03 (0.68, 1.56) | .88 |
| 5 and higher | 0.85 (0.63, 1.16) | 0.31 | 1.12 (0.75, 1.66) | .59 |
| 0 | ref |  | ref |  |
| DoW Affiliation | |  |  |  |
| Navy | 0.93 (0.85, 1.02) | 0.10 | 0.95 (0.84, 1.07) | .36 |
| Air Force | 1.04 (0.93, 1.16) | 0.49 | 1.02 (0.89 ,1.17) | .78 |
| Other | 0.79 (0.62, 1.02) | 0.07 | 0.85 (0.62, 1.16) | .30 |
| Army | ref |  | ref |  |
| Education |  |  |  |  |
| Associate/Vocational | 1.29 (1.15, 1.46) | <.0001 | 1.38 (1.17, 1.63) | .0002 |
| Bachelors | 1.04 (0.93, 1.17) | 0.48 | 1.16 (0.99, 1.35) | .06 |
| Higher (e.g. Masters, PhD) | 0.98 (0.89, 1.09) | 0.76 | 1.19 (1.03, 1.39) | .02 |
| High School | ref |  | ref |  |
| Ethnicity |  |  |  |  |
| Hispanic | 1.23 (1.11, 1.37) | <.0001 | 1.27 (1.10, 1.46) | .001 |
| Non-Hispanic | ref |  | ref |  |
| Sex |  |  |  |  |
| Male | 0.75 (0.70, 0.81) | <.0001 | 0.66 (0.59, 0.74) | <.0001 |
| Female | ref |  | ref |  |
| Military |  |  |  |  |
| Retired Military | 0.76 (0.68, 0.85) | <.0001 | 0.70 (0.58, 0.85) | .0004 |
| Dependent | 0.98 (0.88, 1.10) | 0.77 | 0.70 (0.58, 0.84) | .0002 |
| Active Duty | ref |  | ref |  |
| Race |  |  |  |  |
| Black | 1.19 (1.06, 1.34) | 0.004 | 1.20 (1.03, 1.41) | .02 |
| Asian | 1.19 (1.02, 1.39) | 0.03 | 1.28 (1.05, 1.57) | .02 |
| Other | 1.06 (0.87, 1.29) | 0.57 | 0.97 (0.74, 1.26) | .80 |
| Multiple | 1.39 (1.18, 1.64) | <.0001 | 1.32 (1.06, 1.65) | .01 |
| White | ref |  | ref |  |
| Season |  |  |  |  |
| 2018-2019 | 0.62 (0.52, 0.74) | <.0001 | 0.68 (0.54, 0.85) | .0009 |
| 2019-2020 | 1.07 (0.98, 1.16) | 0.13 | 1.15 (1.03, 1.28) | .02 |
| 2020-2021 | 0.31 (0.27, 0.36) | <.0001 | 0.27 (0.23, 0.31) | <.0001 |
| 2021-2022 | ref |  | ref |  |
| Smoking |  |  |  |  |
| Current | 0.98 (0.81, 1.19) | 0.85 | 1.05 (0.82, 1.36) | .68 |
| Former | 1.14 (1.02, 1.28) | 0.02 | 1.26 (1.08, 1.48) | .003 |
| Non-smoker | ref |  | ref |  |

Abbreviation: CCI, Charlson Comorbidity Index; DoW, Department of War; IRR, incidence rate ratio; aIRR, adjusted incidence rate ratio; ref, reference category

^a^Crude models are bivariate models between each variable and the outcome (number of ILI episodes reported).

**Supplemental Table 3.** Crude and adjusted linear regression model estimates to identify association between covariates and ILI episode duration reported among PAIVED study participants selected for analysis

| **Characteristic** | **Crude^a^** | | **Adjusted** | |
| --- | --- | --- | --- | --- |
|  | **estimate (95% CI)** | **p** | **estimate (95% CI)** | **p** |
| BMI |  |  |  |  |
| Overweight | -0.11 (-0.90, 0.68) | .78 | -0.15 (-0.95, 0.65) | .71 |
| Obese / Severely Obese | 0.96 (0.08, 1.83) | .03 | 0.38 (-0.58, 1.33) | .44 |
| Normal / Underweight | ref |  | ref |  |
| Activity Level | |  |  |  |
| Sedentary | 0.24 (-0.74, 1.21) | .63 | -0.57 (-1.59, 0.44) | .27 |
| Active | ref |  | ref |  |
| Age | 0.07 (0.04, 0.10) | <.0001 | 0.05 (0.02, 0.09) | .01 |
| CCI |  |  |  |  |
| 1-2 | 1.35 (0.23, 2.46) | .02 | 0.28 (-0.89, 1.45) | .64 |
| 3-4 | 0.33 (-2.36, 3.02) | .81 | -1.24 (-3.98, 1.49) | .37 |
| 5 and higher | 1.26 (-1.36, 3.88) | .34 | -0.42 (-3.09, 2.26) | .76 |
| 0 | ref |  | ref |  |
| DoW Affiliation | |  |  |  |
| Navy | -1.31 (-2.08, -0.54) | .0008 | -0.93 (-1.74, -0.12) | .02 |
| Air Force | -0.94 (-1.86, -0.02) | .04 | -0.90 (-1.81, 0.01) | .05 |
| Other | -0.03 (-2.20, 2.14) | .98 | -1.29 (-3.46, 0.88) | .24 |
| Army | ref |  | ref |  |
| Education |  |  |  |  |
| Associate/Vocational | 0.54 (-0.51 1.60) | .31 | -0.24 (-1.33, 0.85) | .67 |
| Bachelors | -0.29 (-1.27 0.69) | .56 | -1.03 (-2.06, 0.01) | .05 |
| Higher (e.g. Masters, PhD) | -0.02 (-0.90 0.86) | .96 | -0.56 (-1.59, 0.46) | .28 |
| High School | ref |  | ref |  |
| Ethnicity |  |  |  |  |
| Hispanic | 0.20 (-0.71, 1.12) | .66 | -0.08 (-1.01, 0.86) | .87 |
| Non-Hispanic | ref |  | ref |  |
| Sex |  |  |  |  |
| Male | -1.10 (-1.76, -0.43) | .0013 | -1.27 (-2.01, -0.53) | .001 |
| Female | ref |  | ref |  |
| Military |  |  |  |  |
| Retired Military | 1.90 (0.92, 2.88) | .0001 | -0.03 (-1.33, 1.27) | .97 |
| Dependent | 1.81 (0.86, 2.76) | .0002 | -0.34 (-1.52, 0.84) | .57 |
| Active Duty | ref |  | ref |  |
| Race |  |  |  |  |
| Black | 1.12 (0.08, 2.15) | .04 | 0.62 (-0.44, 1.67) | .25 |
| Asian | 0.88 (-0.45, 2.22) | .20 | 1.35 (0.02, 2.69) | .05 |
| Other | 0.96 (-0.78, 2.69) | .28 | 0.66 (-1.09, 2.40) | .46 |
| Multiple | 1.02 (-0.44, 2.48) | .17 | 1.03 (-0.42, 2.48) | .16 |
| White | ref |  | ref |  |
| Season |  |  |  |  |
| 2018-2019 | 5.47 (3.98, 6.96) | <.0001 | 4.48 (2.90, 6.06) | <.0001 |
| 2019-2020 | 1.62 (0.90, 2.33) | <.0001 | 1.50 (0.77, 2.24) | <.0001 |
| 2020-2021 | -0.49 (-1.69, 0.70) | .42 | -0.49 (-1.69, 0.70) | .42 |
| 2021-2022 | ref |  | ref |  |
| Smoking |  |  |  |  |
| Current | 1.06 (-0.64, 2.76) | .22 | 0.83 (-0.88, 2.54) | .34 |
| Former | 1.46 (0.47, 2.46) | .004 | 0.97 (-0.04, 1.99) | .06 |
| Non-smoker | ref |  | ref |  |

Abbreviation: CCI, Charlson Comorbidity Index; DoW, Department of War; ref, reference category; CI, confidence interval

^a^Crude models are bivariate models between each variable and the outcome (ILI episode duration).

**Supplemental Table 4.** Crude and adjusted negative logistic regression model estimates to identify association between BMI and moderate-to-severe influenza-like-illness (ILI) symptoms (maximum Flu-PRO score >2) reported among PAIVED study participants

|  |  | **Crude** |  | **Adjusted^a^** |  |
| --- | --- | --- | --- | --- | --- |
| **Domain** | **BMI^b^** | **OR (95% CI)** | **p** | **aOR (95% CI)** | **p** |
| Eye | Overweight | 1.01 (0.75, 1.38) | .92 | 0.97 (0.72, 1.31) | .83 |
|  | Obese / Severely Obese | 1.47 (1.09, 1.99) | .01 | 1.18 (0.85, 1.64) | .33 |
| Gastrointestinal | Overweight | 0.87 (0.57, 1.33) | .52 | 1.00 (0.66, 1.51) | .98 |
|  | Obese / Severely Obese | 1.46 (0.96, 2.22) | .08 | 1.64 (1.01, 2.70) | .04 |
| Nose | Overweight | 0.99 (0.89, 1.10) | .83 | 0.99 (0.89, 1.11) | .91 |
|  | Obese / Severely Obese | 1.07 (0.96, 1.20) | .24 | 1.10 (0.97, 1.25) | .14 |
| Respiratory | Overweight | 1.07 (0.87, 1.32) | .50 | 1.09 (0.88, 1.33) | .43 |
|  | Obese / Severely Obese | 1.45 (1.18, 1.78) | .0004 | 1.33 (1.05, 1.67) | .02 |
| Sense^c^ | Overweight | 1.09 (0.74, 1.60) | .66 | . | . |
|  | Obese / Severely Obese | 1.44 (0.96, 2.16) | .07 | . | . |
| Systemic | Overweight | 1.07 (0.88, 1.29) | .51 | 1.12 (0.93, 1.35) | .24 |
|  | Obese / Severely Obese | 1.29 (1.06, 1.58) | .01 | 1.32 (1.06, 1.63) | .01 |
| Throat | Overweight | 0.97 (0.84, 1.12) | .68 | 0.99 (0.86, 1.14) | .93 |
|  | Obese / Severely Obese | 1.20 (1.04, 1.39) | .01 | 1.20 (1.02, 1.40) | .03 |
| Total (w/o sense) | Overweight | 0.92 (0.66, 1.28) | .63 | 0.95 (0.68, 1.31) | .75 |
|  | Obese / Severely Obese | 1.56 (1.13, 2.16) | .01 | 1.41 (0.98, 2.03) | .07 |

Abbreviation: BMI, body mass index; OR, odds ratio; aOR, adjusted odds ratio; ref, reference category

^a^Models adjusted for activity level, age at enrollment, comorbidity, DoW affiliation, education, ethnicity, sex, military status, race, season, and smoking status.

^b^Normal/underweight is the reference category

^c^The adjusted model for sense domain failed to converge; therefore, we did not present the adjusted odds ratio in this table.

**Supplemental Table 5.** Adjusted model estimates from sensitivity analyses for all outcomes (ILI incidence, ILI duration, Flu-PRO domains, and lab-confirmed respiratory viruses) among PAIVED study participants. Parsimonious models included BMI and two covariates selected via stepwise regression

| **ILI incidence**^a^ | **BMI** | **aIRR (95% CI)** | **P** |
| --- | --- | --- | --- |
|  | Overweight | 1.16 (1.04, 1.31) | .01 |
|  | Obese | 1.46 (1.29, 1.67) | <.0001 |
| **ILI duration (days)**^b^ | **BMI** | **Estimate (95% CI)** | **P** |
|  | Overweight | -0.29 (-0.58, 0.00) | .46 |
|  | Obese | 0.31 (0.00, 0.62) | .50 |
| **Flu-PRO**^c^ | **BMI** | **aRR (95% CI)** | **P** |
| Eyes | Overweight | 1.04 (0.74, 1.46) | .47 |
|  | Obese | 1.37 (0.96, 1.96) | .08 |
| Gastrointestinal | Overweight | 0.85 (0.54, 1.33) | .81 |
|  | Obese | 1.38 (0.86, 2.21) | .18 |
| Nose | Overweight | 0.97 (0.79, 1.20) | .52 |
|  | Obese | 1.10 (0.87, 1.40) | .43 |
| Respiratory | Overweight | 1.09 (0.84, 1.41) | .64 |
|  | Obese | 1.49 (1.12, 1.97) | .01 |
| Systemic | Overweight | 1.06 (0.83, 1.36) | .56 |
|  | Obese | 1.25 (0.95, 1.64) | .10 |
| Throat | Overweight | 0.94 (0.75, 1.17) | .59 |
|  | Obese | 1.21 (0.95, 1.55) | .13 |
| Total (w/o sense) | Overweight | 0.91 (0.63, 1.30) | .57 |
|  | Obese | 1.42 (0.97, 2.07) | .07 |
| **Lab-confirmed Virus**^d^ | **BMI** | **aOR (95% CI)** | **P** |
| Influenza (any)^e^ | Overweight | 0.97 (0.62, 1.51) | .89 |
|  | Obese | 0.72 (0.41, 1.24) | .23 |
| A^e^ | Overweight | 1.00 (0.58, 1.73) | .99 |
|  | Obese | 0.68 (0.35, 1.32) | .25 |
| A/H1N^e^ | Overweight | 0.83 (0.42, 1.64) | .59 |
|  | Obese | 0.58 (0.26, 1.30) | .18 |
| A/H3N2^e^ | Overweight | 1.30 (0.42, 4.03) | .66 |
|  | Obese | 1.14 (0.29, 4.53) | .86 |
| A/untyped^e^ | Overweight | 1.60 (0.30, 8.39) | .57 |
|  | Obese | 0.72 (0.10, 5.47) | .76 |
| B^e^ | Overweight | 0.99 (0.48, 2.06) | .98 |
|  | Obese | 0.88 (0.34, 2.28) | .79 |
| Human Coronavirus | Overweight | 1.10 (0.76, 1.58) | .62 |
|  | Obese | 1.17 (0.77, 1.77) | .46 |
| HMPV^e^ | Overweight | 0.60 (0.29, 1.24) | .17 |
|  | Obese | 0.90 (0.42, 1.92) | .78 |
| PIV^e^ | Overweight | 1.13 (0.40, 3.21) | .82 |
|  | Obese | 1.92 (0.66, 5.58) | .23 |
| Rhino/enterovirus | Overweight | 0.81 (0.62, 1.06) | .12 |
|  | Obese | 0.95 (0.70, 1.29) | .74 |
| RSV^e^ | Overweight | 1.53 (0.76, 3.08) | .24 |
|  | Obese | 1.21 (0.54, 2.72) | .64 |
| SARS-CoV-2 | Overweight | 1.06 (0.79, 1.44) | .70 |
|  | Obese | 0.74 (0.52, 1.06) | .10 |
| Any respiratory virus | Overweight | 1.03 (0.83, 1.26) | .80 |
|  | Obese | 0.90 (0.71, 1.15) | .40 |

Abbreviation: BMI, body mass index; aIRR, adjusted incidence rate ratio; aRR, adjusted risk ratio; aOR, adjusted odds ratio; HMPV, Human metapneumovirus; PIV, RSV, Respiratory syncytial virus; parainfluenza virus

^a^Model adjusted for season and sex.

^b^Model adjusted for season and age at enrollment.

^c^Models adjusted for season and education level.

^d^Models adjusted for age at enrollment and ethnicity.

^e^For virus outcomes with limited cases, only parsimonious models were used in the main analysis

**
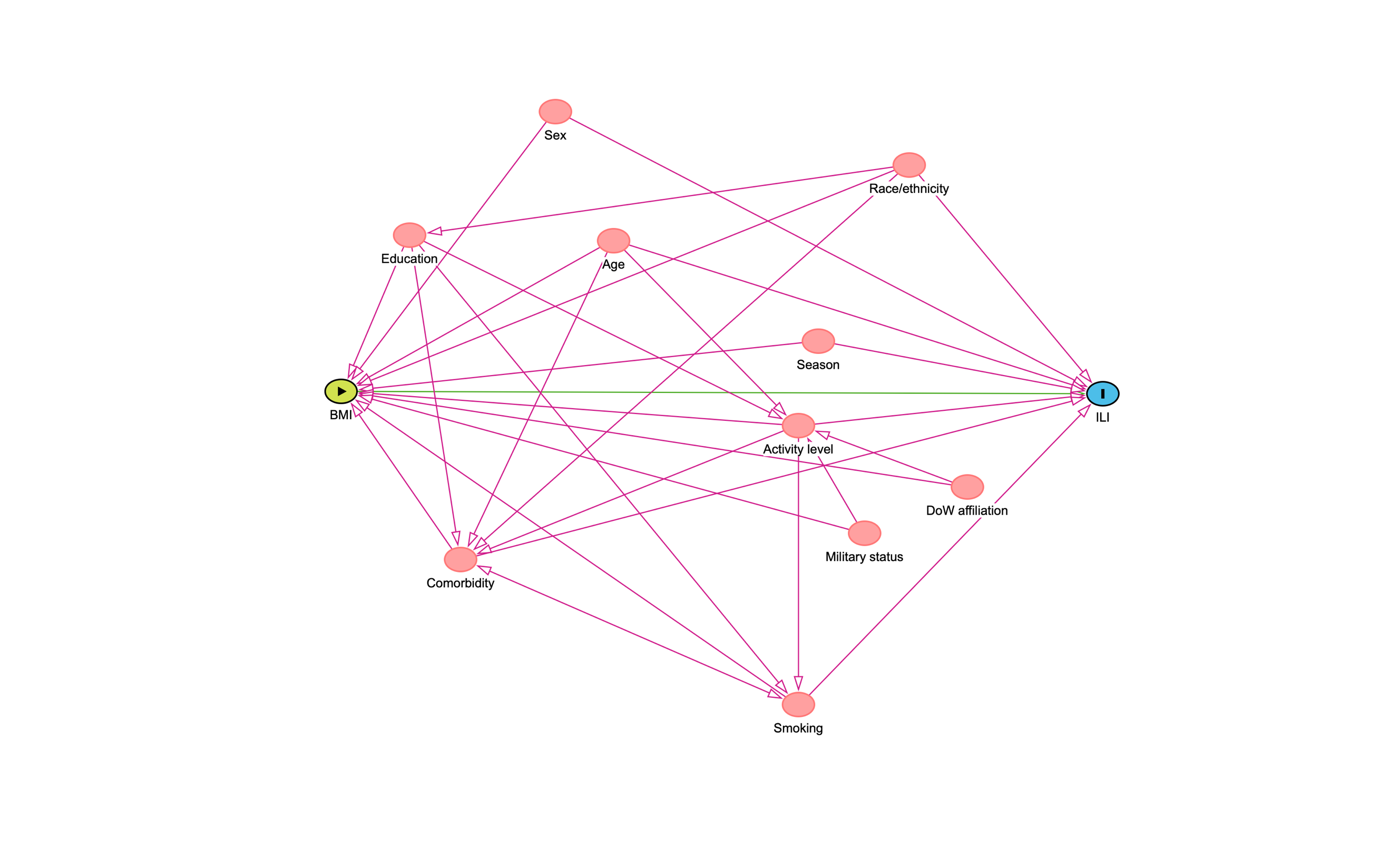
**

**Supplemental Figure 1.** Directed Acyclic Graph (DAG) depicting the conceptual framework for associations between BMI and ILI. DAG illustrating assumed confounding structure was created using DAGitty (Textor et al., 2016).


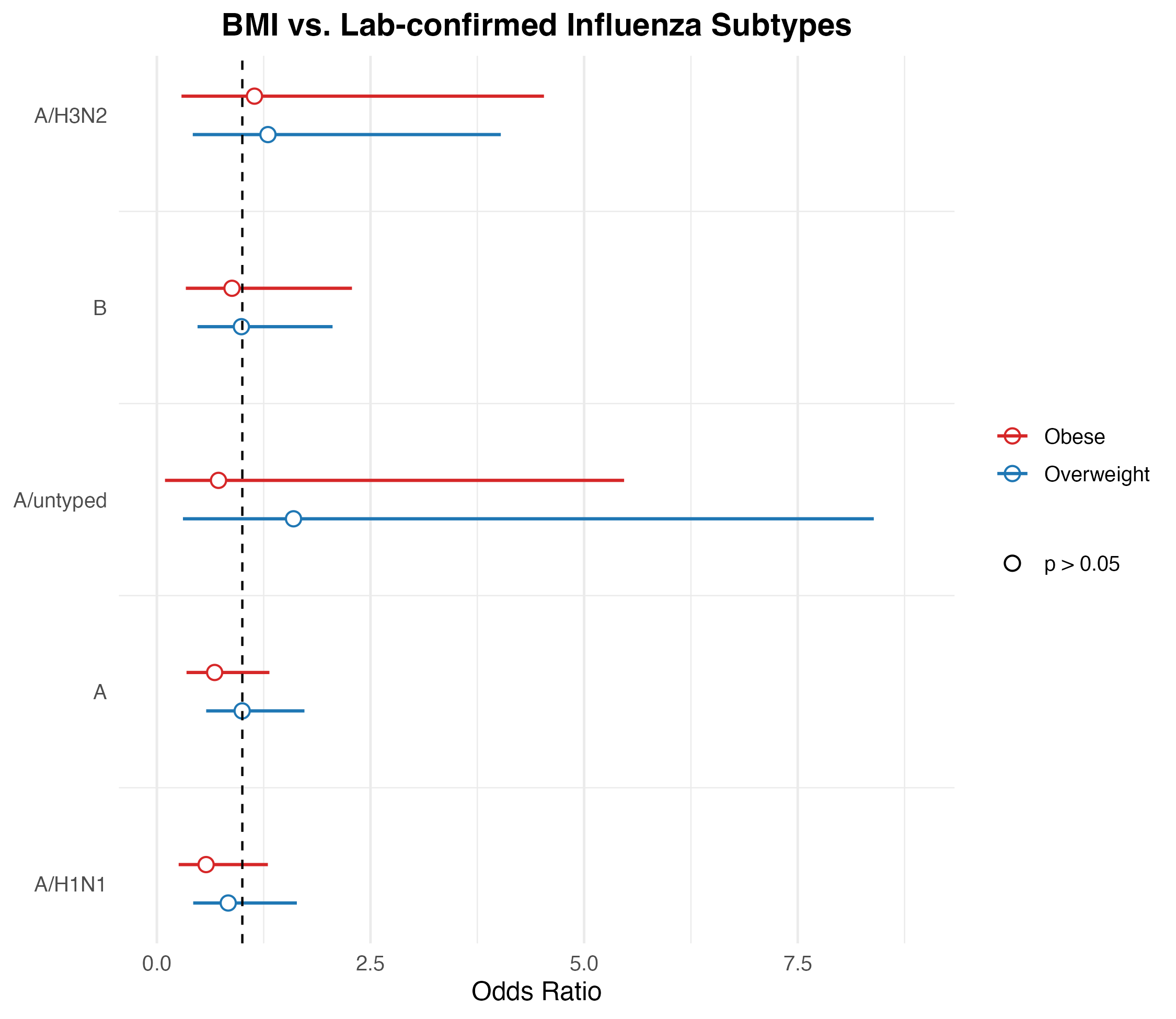


**Supplemental Figure 2.** Adjusted odds ratio (aOR) of lab-confirmed respiratory infection by BMI category (using normal/underweight BMI as reference) generated from logistic regression models. Analyses were done separately for each type of virus.
